# Editorial: Congenital Adrenal Hyperplasia, Unresolved Issues and Implications on Clinical Management

**DOI:** 10.3389/fendo.2020.00170

**Published:** 2020-03-31

**Authors:** Sarantis Livadas, Constantine A. Stratakis, Djuro Macut

**Affiliations:** ^1^Endocrine Unit, Metropoltan Hospital, Athens, Greece; ^2^Medical Genetics, Pediatrics, Pediatric Endocrinology, Bethesda, MD, United States; ^3^Clinic of Endocrinology, Diabetes and Metabolic Diseases, Faculty of Medicine, University of Belgrade, Belgrade, Serbia

**Keywords:** adrenals, PCOS (polycystic ovarian syndrome), cortisol, andrognes, insulin resisitance

Congenital Adrenal Hyperplasia (CAH) constitutes a group of autosomal recessive disorder, arising from mutations in the genes regulating steroidogenesis. Mutations in the CYP21A2 gene account for ~95% of CAH cases and most affected individuals are compound heterozygotes arising from inheritance of different paternal and maternal mutant alleles of this gene. However, there is an increasing incidence of true homozygosity due to consanguineous marriage. The incidence of classical CAH in western populations is about 1:40.000 and with a carrier frequency of 1 in 50. Conversely, the prevalence of non-classical CAH, which remains relatively under-diagnosed, may be as high as 1:100-1:1,000 and even higher (1–20) among Hispanics, Yugoslavs, and Ashkenazi Jews.

The discrimination of CAH to Classical and Non-Classical forms is based on the degree of 21-hydroxylase deficiency. Namely, in classic CAH, inactivating mutations of the gene lead to salt wasting due to the absence of both aldosterone and cortisol production, whereas excess adrenal androgens causing signs of virilisation. On the other hand, in Non-Classical CAH, partial enzyme activity is sufficient for cortisol and aldosterone secretion, but increased androgen production are related to variable phenotypes, which may occur prepubertally, during adolescence or later in adulthood. Nevertheless, CAH is better to be considered as a continuum of diseases due to phenotypic variability through time.

Although the Synacthen test is a prerequisite in differential diagnosis, the genotyping of the affected patient is required for confirmation of the diagnosis. Furthermore, in difficult cases, accurate molecular diagnosis essential for optimal genetic counseling and during preparation for pregnancy. On the other hand, genotyping is a challenging process due to the occurrence of both a gene and a highly homologous pseudogene. Nevertheless, as is meticulously explained in their article “*The Complexities in Genotyping of Congenital Adrenal Hyperplasia”* by Pignatelli et al., novel tools are improving the chances of a correct diagnosis and better understanding of the underlying mechanisms of the disease. The new opportunities now provided by genotyping, however, also entail new complexities (Pignatelli et al.). Beyond the 10 classic pathogenic variants usually examined in most laboratories, achievement of an in-depth analysis of 21OH-deficiency cases will today involve complete sequencing of the entire gene and identification of gene duplications, which occur frequently and may lead to false positive cases. Moreover, since gene conversions can include several pathogenic variants, it cannot be established with absolute certainty whether both alleles are affected without studying parental DNA samples. The treating clinician should therefore be aware of the challenges, but also of the new possibilities available, and of both the precision of molecular techniques and their difficulties. Most importantly, a suspected diagnosis of NCCAH should certainly not be ruled out until and unless full sequencing of the CYP21A2 gene and the abovementioned procedures have been applied by an experienced and up-to-date laboratory.

Another issue in the diagnosis of CAH is the lack of significant genotype-phenotype association (~80%), an unusual phenomenon in monogenic diseases. Indeed, in the case of thalassaemia or cystic fibrosis, the phenotype is closely related to a genetic defect. In line with these data, in their article “*Genotype Is Associated to the Degree of Virilization in Patients With Classic Congenital Adrenal Hyperplasia”*, Neocleous et al. analyzed the impact of different molecular defects on the degree of virilisation in their cohort of CAH patients. Interestingly, by applying state-of-the-art techniques, they observed disorders ranging from complete male virilisation to clitoromegaly in female newborns carrying CYP21A2 mutations, thus emphasizing the need for full genotyping of patients with the disease regardless of the clinical presentation, especially in cases requiring genetic counseling or involving future fecundity (Neocleous et al.).

Another very important but undervalued issue is the fact that patients with NCCAH are at increased risk of metabolic derangements. Indeed, in females, there is substantial evidence showing a direct link between hyperandrogenaemia and insulin resistance. Furthermore, a significant percentage of patients are on chronic treatment with glucocorticoids, oral contraceptive pills, and/or antiandrogens, therapeutic regimens which induce an unfavorable metabolic profile. Consequently, increased incidence of dysglycaemia, dyslipidaemia, hypertension, fatty liver, and metabolic syndrome is anticipated in this hyperandrogenic population. However, given that the available data evaluating this association are to date limited, the recent publication of three articles aiming at elucidating this relationship is of major importance. First, de Vries et al., in their article “*Obesity and Cardiometabolic Risk Factors in Children and Young Adults With Non-classical 21-Hydroxylase Deficiency”*, assessed the metabolic profile of a large number of adolescents with the disease. Of note, 66% of them were on treatment with glucocorticoids. Also of special interest is the authors' observation that patients with NCCAH do not differ from their normal peers as regards obesity status and metabolic profile, irrespective of glucocorticoid treatment.

Regarding cardiovascular risk in patients with CAH, a detailed review entitled “*Cardiovascular Health in Children and Adolescents With Congenital Adrenal Hyperplasia Due to 21-Hydroxilase Deficiency”* was carried out by Improda et al.. Their working hypothesis was that, based on the fact that a cluster of cardiovascular (CV) risk factors have been documented in adults with CAH, and it is highly likely that children and adolescents are prone to develop a similar sequela. As concerns obesity, though few data are as yet available on NCCAH patients, no significant differences were found between classic and non-classic forms. Furthermore, concerning insulin resistance, the available data indicate that both treated and untreated NCCAH patients display reduced insulin sensitivity. Of note, in one study it was observed that patients with NCCAH displayed more pronounced alterations in glucose metabolism compared to patients with classic forms. Taking the above as a whole, it could be suggested that prolonged exposure to androgen excess may contribute to an adverse metabolic profile. Due to the shortage of data, assessment of CV risk factors, such as adipokines, inflammatory markers, and homocysteine, as well as of heart function, was necessary in the NCCAH patients of the above study. Generally speaking, however, young patients with the classic form of the disease exhibit an unbalanced profile of several CV markers associated with vascular dysfunction and increased intramedial thickness of the carotid arteries, as well as with impaired exercise performance. The authors conclude that CAH may be associated with early markers of CV morbidity. Thus, lifestyle counseling and periodic assessment of blood pressure at all ages should be recommended in the management of CAH children, with regular monitoring of CV markers tailored to the individual patient's needs.

Adopting a different approach, Macut et al. analyzed the incidence of growth, obesity, insulin resistance, and bone mineral density in patients with NCCAH at different ages and compared them with subjects with the classic form of the disease. In their article entitled “*Metabolic Perspectives for Non-classical Congenital Adrenal Hyperplasia With Relation to the Classical Form of the Disease”*, the authors discuss the metabolic consequences in these patients and describe the therapeutic effects of the different drug regimens, while pointing to the lack of longitudinal follow-up data (Macut et al.). With respect to growth, no difference was found between patients with NCCAH and the normal population, since almost normal final height was observed. It was however noted that adult NCCAH patients are prone to develop metabolic consequences, since many of them presented higher rates of obesity, insulin resistance, and CV risk factors. Finally, decreased bone mineral density and osteoporosis were found in some cases, this possibly being partially attributable to gender as well as to the type and dose of glucocorticosteroids applied.

NCCAH has been detected in women with androgen excess, with varying prevalence (1–10%), while both clinical and hormonal findings in females with NCCAH overlap with those of other hyperandrogenic entities, such as polycystic ovary syndrome (PCOS), hence causing diagnostic difficulties. This important issue has been extensively reviewed by Papadakis et al. in their article entitled “*Polycystic Ovary Syndrome and NC-CAH: Distinct Characteristics and Common Findings. A Systematic Review*”. Specifically, the article discusses the differences in the genetics and pathophysiology that regulate these disorders. Meanwhile, however, such common findings as hirsutism, hyperandrogenaemia, polycystic morphology, and pregnancy complications, as well as metabolic disturbances and mood disturbances, are also reviewed, thereby emphasizing that the two entities are also characterized by small differences. Furthermore, the need for Synacthen testing in the differential diagnosis is presented, while there is additionally a thorough examination of the various therapeutic approaches adopted in accordance with the underlying disorder.

In the same line of thought, Livadas and Bothou, in their article “*Management of the Female With Non-classical Congenital Adrenal Hyperplasia (NCCAH): A Patient-Oriented Approach”*, present the best possible approach to the female patient with NCCAH based on the patient's prevailing symptoms at the specific age of presentation (Livadas and Bothou). First, they describe the diverse phenotype of the disease in childhood, during adolescence, or later in adult life, this followed by their recommendations for management. Specifically concerning the treatment decision, they emphasize that treatment (1) should be individualized, (2) is not always indicated, and (3) has to be modified according to the patient's evolving needs. Also described are the therapeutic options for female patients treated from childhood, those who presented the first signs and symptoms during adolescence, and those with hyperandrogenic symptoms post-treatment discontinuation. A detailed discussion on the current therapeutic regimens of, inter alia, glucocorticoids, oral contraceptive pills, and antiandrogens, is provided. Furthermore, the major issue of subfertility, usually encountered in females with NCCAH, is systematically analyzed. Advice on molecular testing of the prospective father is based on the potential mother's genotype. The authors propose a tailor-made approach, incorporating a smooth transition of the patient's management once she is referred from the pediatric to the adult endocrinologist, while underlining that she will require symptom-oriented treatment throughout her life.

Another hot topic concerning the pathophysiology and management of women with hyperandrogenic disorders is the impact of vitamin D deficiency. Indeed, dysregulation of this pleiotropic hormone has been implicated in subclinical inflammation, insulin resistance, and hyperandrogenaemia, all factors that characterize the PCOS phenotype. Yu-Ming et al., in their article “*Association Between Vitamin D Receptor Gene Polymorphisms and Polycystic Ovary Syndrome Risk: A Meta-Analysis”* provide some of the current data on the effect of vitamin D and its receptor polymorphisms on PCOS pathophysiology and, based on their meta-analysis, they suggest that vitamin D receptor gene polymorphisms contribute to PCOS development (Yu-Ming et al.).

Finally, subfertility is a common problem in women suffering from CAH, with many of those suffering from NCCAH often being diagnosed for the first time in a fertility clinic. It is well known that the increased progesterone concentrations usually encountered in this population alter endometrial receptivity and tubal motility and lead to ovulation disorders. In the review “*Assisted Reproduction in Congenital Adrenal Hyperplasia”* by Chatziaggelou et al., these issues are very well-analyzed and an optimal approach is suggested. The authors suggest that administration of an adequate substitution dose of glucocorticoids leads not only to successful assisted reproduction treatment but also, in many cases, to spontaneous pregnancy. Hydrocortisone is today the gold standard treatment, since it restores ovarian function, ovulation, and endometrial receptivity. They recommend that in the event of a pregnancy involving a fetus suspected of having CAH, delivery should ideally be managed by an expert, multidisciplinary team, including a gynecologist, an endocrinologist, and a pediatrician, in a tertiary hospital.

In conclusion in this Editorial illuminating the articles of this series, we have sought to provide the clinician with the current knowledge regarding several aspects of the management of patients with NCCAH. The latter disorder represents a particular form of CAH that is characterized by specificities in clinical presentation, diagnosis, therapeutic approach, and metabolic outcomes. Though concerning a less severe form of CAH, therapeutic management of NCCAH patients remains a challenge, and current treatment regimens do not always allow optimal biochemical control, while overexposure to glucocorticoids as well as to androgen excess may contribute to the development of metabolic and cardiovascular abnormalities. Therefore, we strongly recommend a patient-oriented approach, based on each patient's individual needs, and long-term follow-up.

## Author Contributions

SL wrote the editorial. CS and DM edited the manuscript.

### Conflict of Interest

The authors declare that the research was conducted in the absence of any commercial or financial relationships that could be construed as a potential conflict of interest.

